# Acai Oil-Based Organogel Containing Hyaluronic Acid for Topical Cosmetic: In Vitro and Ex Vivo Assessment

**DOI:** 10.3390/pharmaceutics16091195

**Published:** 2024-09-11

**Authors:** Suellen Christtine da Costa Sanches, Lindalva Maria de Meneses Costa Ferreira, Rayanne Rocha Pereira, Desireé Gyles Lynch, Ingryd Nayara de Farias Ramos, André Salim Khayat, José Otávio Carrera Silva-Júnior, Alessandra Rossi, Roseane Maria Ribeiro-Costa

**Affiliations:** 1 Laboratory of Pharmaceutical Nanotechnology, Faculty of Pharmaceutical Sciences, Federal University of Pará, Belém 66075-110, Brazil; suellen.sanches@yahoo.com.br (S.C.d.C.S.); lindalva.costa.ferreira@ics.ufpa.br (L.M.d.M.C.F.); 2 Institute of Collective Health, Federal University of Western Para, Santarém 68135-110, Brazil; rayannerocha@yahoo.com; 3 School of Pharmacy, College of Health Sciences, University of Technology, Jamaica, 237 Old Hope Road, Kingston 6, Jamaica; 4 Oncology Research Center, Federal University of Pará, Belém 66075-110, Brazil; ingrydramos@yahoo.com.br (I.N.d.F.R.); khayatas@gmail.com (A.S.K.); 5 Pharmaceutical and Cosmetic R&D Laboratory, Faculty of Pharmaceutical Sciences, Federal University of Pará, Belém 66075-110, Brazil; carrera@ufpa.br; 6 Department of Food and Drug, University of Parma, Parco Area delle Scienze 27/A, 43124 Parma, Italy; alessandra.rossi@unipr.it

**Keywords:** acai oil, 12-hydroxystearic acid, hyaluronic acid, organogel, topical cosmetic

## Abstract

Organogels are semi-solid pharmaceutical forms whose dispersing phase is an organic liquid, for example, an oil, such as acai oil, immobilized by a three-dimensional network formed by the gelling agent. Organogels are being highlighted as innovative release systems for cosmetic active ingredients such as hyaluronic acid for topical applications. Acai oil was evaluated for its physicochemical parameters, fatty acid composition, lipid quality index, spectroscopic pattern (Attenuated total reflectance Fourier Transform Infrared Spectroscopy), thermal behavior, total phenolic, total flavonoids, and total carotenoids and β-carotene content. The effectiveness of the organogel incorporated with hyaluronic acid (OG + HA) was evaluated through ex vivo permeation and skin retention tests, in vitro tests by Attenuated total reflectance Fourier Transform Infrared Spectroscopy and Differential Scanning Calorimetry. The physicochemical analyses highlighted that the acai oil exhibited quality standards in agreement with the regulatory bodies. Acai oil also showed high antioxidant capacity, which was correlated with the identified bioactive compounds. The cytotoxicity tests demonstrated that the formulation OG + HA does not release toxic substances into the biological environment that could impede cell growth, adhesion, and efficacy. In vitro and ex vivo analyses demonstrated that after 6 h of application, OG + HA presented a high level of hydration, thermal protection and release of HA. Thus, it can be concluded that the OG + HA formulation has the potential for physical–chemical applications, antioxidant quality, and potentially promising efficacy for application in the cosmetic areas.

## 1. Introduction

The industrial sector is increasingly seeking safer, biocompatible, and effective cosmetic formulations; a proposal is the combination of vegetable oils that have antioxidant characteristics with another, preferably natural, active ingredient in order to provide different functions and associated effects, such as preventing premature aging, providing elasticity, firmness, hydration, among other benefits for the skin [1]. In this context, the biological properties of natural Amazonian oils are distinguished by their potential for moisturizing, emulsifying, and for containing antioxidant bioactive substances that are extremely important for reducing the negative effects of free radicals generated by the natural oxidative processes of metabolism, representing one of the main causes of premature aging. Thus, the Amazon region stands out for the potential that its flora can offer to the world, among which are the palm trees of the Arecaceae family, one of the main representatives of which is the acai [2,3].

Acai (Euterpe oleracea Martins), a palm tree native to the Amazon, is mainly used for the consumption of the pulp and the juice extracted from its fruits, which are characterized by a high nutritional value. The processing of the acai generates seeds as a by-product, which recent studies have shown to have considerable oil content. This oil can be used as an alternative energy source and for the manufacturing of medicines and cosmetics [2,3].

Acai oil is a new cosmetic active ingredient originating from the Amazon Rainforest. Its structure is mainly composed of anthocyanins, flavonoids, and carotenoids, which are widely used by the cosmetic industry as antioxidants to prevent skin aging by promoting cellular metabolism and reducing inflammatory processes [2,3]. Antioxidants are substances that protect cell membranes against the generation of oxidative stress in tissues and are characterized by their ability to neutralize toxic oxygen molecules and free radicals. These reactive oxygen species (ROS) are continuously produced during cellular metabolism, as are other free radicals, which originate when the electron is alone in an orbit, i.e., the atom or molecule is not coupled [2].

The trend in the cosmetics industry is to develop innovative formulations that contain more effective and stable vegetable oils to solve esthetic problems by diversifying the available options, which range from traditional moisturizing products to those that go beyond cosmetic and esthetic purposes to preventing and/or combatting premature aging. Among the products aimed at this purpose, preparations using organogels (OGs) technology are distinguished [4]. OGs are viscoelastic materials composed of gelling agents and a nonpolar liquid phase (natural oil), forming semisolid systems, in which the oily phase is immobilized in the self-sustaining three-dimensional network of the gelling agent. OGs are obtained from the mechanism of trapping the solubilized oil molecules in the three-dimensional fibrous network formed by the self-assembled gel structure during the heating phase. Then, the formulation is cooled below the gelation transition temperature to obtain the OG system [5,6].

OG, obtained by low-molecular-weight gelling agents (LMWGAs), is used in cosmetic productions due to its ability to form a gel structure even with a low percentage of structuring agent, typically 2% (*w*/*w*). In addition, LMWGAs have a series of advantages in the application of OG, such as the solid fibrillar matrix improving their mechanical properties, which consequently increases the encapsulation rate of lipophilic or amphiphilic drugs [3]. For example, the LMWGA, 12-hydroxystearic acid (12HSA), was used to prepare an OG [5].

Active pharmaceutical ingredients (API) to be encapsulated in the OG can be hydrophilic or lipophilic for topical (dermal) or transdermal administration [3]. Hyaluronic acid (HA) is a highly hydrophilic natural moisturizing compound that is utilized in most rejuvenation facial treatments because, due to its gelatinous and thick consistency, elasticity, and degree of hydration, it acts as a space filler, contributing to the improvement of the skin structure and elasticity, removing wrinkles, enhancing and restoring facial volume, creating lip volume, smoothing expression lines, fibroblast, and scars [7].

HA has significant cosmetic efficacy in resolving different skin defects, such as premature skin aging, nasolabial folds, and wrinkles [8]. In order to investigate these effects, HA has been used in different forms, i.e., creams, serums, gels, lotions, intradermal filler injections, and facial fillers. The effects of HA have been associated with its ability to promote facial rejuvenation, collagen stimulation, and tissue augmentation [5,8,9].

The administration of cosmetic products to the skin can be divided into two categories: local and/or systemic effects. Topical products treat diseases in the different layers of the skin or in cutaneous appendages; therefore, their efficacy will depend on the retention time of the drug and its concentration in these sites and the hydrodynamic diameter of the nanostructure [10,11,12].

In a study by Costa Sanches et al. [5], the physicochemical characteristics of the acai-oil-based OG (acai oil and 12HSA) and the OG based on acai oil containing HA (acai oil, 12HSA, and HA) were evaluated, and it was concluded that the OG containing HA (OG + HA) presented promising physicochemical characteristics suitable for cosmetic application. Thus, the current research is a continuation of the previous study to analyze the acai oil in terms of fatty acids and phytochemicals composition and antioxidant activity to determine the quality and authenticity of the oil. Due to the chemical complexity of the acai oil, it is necessary to guarantee the safety of the raw material used in the manufacturing process of the OG in accordance with the regulatory agencies [13]. Moreover, ex vivo studies were carried out to understand the biopharmaceutical behavior of the OG + HA formulation in relation to the penetration and/or permeation of HA in the skin. Furthermore, analytical tests were performed to predict the degree of skin hydration provided by the OG + HA system.

## 2. Materials and Methods

### 2.1. Materials

The acai oil (Lot: H2004410) was obtained from BERACA natural ingredients S/A located in Ananindeua city in the state of Pará located in the Brazilian Amazonian region and stored at −30 °C until analysis. From Sigma-Aldrich (St. Louis, MO, USA), 2,2-azinobis (3-ethylbenzothiazolin-6)-sulfonic acid) (ABTS), 6-Hydroxy-2,5,7,8-tetramethylchroman-2-carboxylic acid (Trolox), linoleic acid, β-carotene, and polyoxyethylene sorbitan monopalmitate (Tween 40) were purchased. Hyaluronic acid (Lot: PS009531/F01) was obtained from a Compounding Pharmacy in Belém (PA, Brasil), and the 12-hydroxystearic structuring agent (Lot: E8050A) was supplied by Alfa Aesar (Tewksbury, MA, USA).

### 2.2. Methods

#### 2.2.1. Physicochemical Characterization of Acai Oil

The physicochemical characteristics of acai oil were assessed by the determination of its refractive index, relative density, kinematic viscosity, free acidity (expressed in the equivalent of oleic acid) peroxide, and saponification. The tests were carried out using the methodologies presented by the American Oil Chemists’ Society (AOCS) [14] and Codex Alimentary Committee [15]. All the analyses were carried out in triplicate.

##### Determination of Fatty Acid Composition

The fatty acid (FA) composition was determined by Gas Chromatography coupled with a Mass Spectrometer (GC-MS) (Shimadzu^®^ Kyoto, Japan). The method for sample preparation was carried out according to the AOCS [14] for methyl esters. The specifications used for injection were as follows: 30.0 m × 0.25 mm column SH-Rtx-5; helium gas as a carrier gas; flow rate, 1.5 mL min^−1^; injection volume, 1 μL (split, partition 1:50). The used temperature ramp was 2 min at 60 °C, heating up to 240 °C at a rate of 10 °C/min, and maintained at that temperature for 8 min. The chromatographic peaks were analyzed by means of Shimadzu GC Solution software (Shimadzu^®^ Corporation, Kyoto, Japan) [14].

##### Attenuated Total Reflectance Fourier Transform Infrared Spectroscopy (ATR-FTIR)

The sample was analyzed under the following conditions: scan 32, resolution 4 cm^−1^, and in the range of 4000 to 600 cm^−1^, using an ATR spectrometer IR Prestige 21 (Shimadzu^®^ Corporation Kyoto, Japan) [16].

##### Thermal Analyses

For thermogravimetric analysis (TG), derived thermogravimetry (DTG), and differential scanning calorimetry (DSC) tests, about 5 mg of acai oil was accurately weighed in a platinum crucible and aluminum crucible, respectively, and the analyses were performed under the following conditions: nitrogen atmosphere (50 mL/min) and heating rate of 10 °C/min in a temperature range from 25 to 550 °C. For TG/DTG and DSC analyses, a TGA-50 thermal analyzer (Shimadzu^®^, Kyoto, Japan) and a DSC-60 plus equipment (Shimadzu^®^, Kyoto, Japan), respectively, were used. The mass loss and enthalpy variation calculations were performed using the TA 60w Shimadzu^®^ program [16].

##### Quantitative Analysis of Total Phenolic Content

A UV/Vis spectrophotometer (Shimadzu UV 1800, Kyoto, Japan) was used for the quantitative analysis of total phenolics in the sample. In total, 100 μL of oil was mixed via a vortex (Vixar, 220 V) in an assay tube with 500 μL of Folin Ciocalteau reagent and 6 mL of purified water. Then, the assay tube was incubated in the dark for 2 min. Subsequently, 2 mL of 10% sodium carbonate solution was added to the assay tube and left for 2 h in the dark, prior the UV analysis at a wavelength of 760 nm for the absorbance value determination. The total phenolics was calculated using a standard curve of gallic acid (5–75 mg/mL) in the mg unit of gallic acid equivalent (mg GAE/100 g) [17].

##### Quantitative Analysis of Flavonoids

A UV/Vis spectrophotometer (Shimadzu UV 1800, Kyoto, Japan) was used for the quantitative analysis of flavonoids in the sample. In total, 800 μL of the oil was mixed via a vortex with 400 μL of 2.5% aluminum chloride in an assay tube and left for 30 min in the dark. Then, the absorbance was measured at a wavelength of 425 nm. The quantification of flavonoids was calculated using a standard curve of rutin (5–30 mg/mL) and expressed in the mg unit of rutin equivalent (mg RUT/100 g) [17].

##### Total Carotenoids Content

This assay was performed using the UV/Vis spectrophotometric method (Shimadzu UV 1800, Kyoto, Japan). In total, 3 g of oil was weighed in a 25 mL beaker and dissolved in 5 mL of hexane. Then, the solution was transferred in a 10 mL volumetric flask and brought to volume with hexane. The analysis was performed at a wavelength of 470 nm, using the specific coefficient for β-carotene (E_o_ = 2592). The analyses were performed in triplicate, and the results were expressed as mg of carotenoids/100 g of oil [18].

##### Quantitative Analysis of β-Carotene

A UV/Vis spectrophotometer (Shimadzu UV 1800, Kyoto, Japan) was used for the quantitative analysis of β-carotene in the sample. In total, 10 mg of oil was solubilized in a 10 mL volumetric flask with hexane. The analysis was performed at a wavelength of 450 nm, using the specific coefficient for β-carotene (E_o_ = 2592). The analyses were performed in triplicate, and the results were expressed as mg of β-carotene/100 g of oil [19].

##### ABTS Free-Radical Activity

The antioxidant activity by the free-radical-capture ABTS was performed by a spectrophotometer (Shimadzu^®^ UV 1800, Kyoto, Japan). In total, 30 μL of the sample was mixed with 3000 μL of an ABTS solution and left in the dark for 6 min, followed by measurement at a wavelength of 734 nm. The antioxidant activity was calculated based on an acai standard curve (100–2000 μM), and the results were expressed in μM Trolox equivalent/g dry weight (TE) [20].

##### DPPH Method

By capturing the DPPH free radical, the antioxidant activity was performed in a UV 1800 spectrophotometer (Shimadzu^®^, Kyoto, Japan) at a wavelength of 515 nm. In a dark environment, 150 µL of the oil was transferred to a test tube. Then, 5.85 mL of the DPPH radical was added and homogenized in a vortex (Vixar, 220 V). After 30 min, the sample was analyzed using the spectrophotometer. The antioxidant activity was calculated based on a standard curve of Trolox (50–2000 µM). The final concentration was expressed as µM Trolox (TE)/g [21].

##### β-Carotene/Linolenic Acid System

The antioxidant activity of the β-carotene/linoleic acid system was performed by using a spectrophotometer (Shimadzu^®^ UV 1800, Kyoto, Japan). The β-carotene/linoleic acid system was prepared by adding 40 μL of linoleic acid, 530 μL of Tween 40, 50 μL of 0.2% of β-carotene solution in chloroform, and 500 μL of chloroform in an Erlemeyer flask, covered with aluminum foil to protect the yellow–orange solution from light to avoid degradation. The mixture was subjected to complete evaporation of chloroform under nitrogen flow by inserting a tube in the Erlemeyer flask. Then, 25 mL of oxygen-treated water was added to the Erlemeyer flask to obtain an absorbance value between 0.6 and 0.7 at a wavelength of 470 nm [22].

In total, 0.4 mL of acai oil was mixed with 5 mL of the previously prepared β-carotene/linoleic acid system. Then, 0.4 mL of the Trolox solution at a concentration of 200 mg/mL was used as a control with 5 mL of the β-carotene/linoleic acid system solution. The test tubes were shaken by vortex and kept in a water bath at 40 °C [22].

The first UV reading was performed at 470 nm after 2 min at 40 °C and then at intervals of 15 min for 2 h. As blank solution, purified water was used. The results were expressed as a percentage of oxidation inhibition. The absorbance value of the sample without the addition of the antioxidant system was considered as 100% oxidation. The decrease in the absorbance value of the oil samples is correlated with the added β-carotene/linoleic acid system: the oxidation percentage of the oil is calculated by subtracting the oxidation percentage value for each diluted oil sample from 100% [22].

#### 2.2.2. Preparation of Organogel Systems

In the preparation of the OG, the following procedure was carried out: The equivalent of 2% (*w*/*w*) of the gelling agent 12-HAS in acai oil was prepared. The mixture (12-HSA and acai oil) was heated to 85 °C for 30 min in a water bath and was stirred for 3 min by a magnetic stirrer. Subsequently, the mixture was cooled to room temperature and left for 24 h to obtain the OG [5].

In the preparation of the acai oil organogel containing hyaluronic acid (OG + HA), the following procedure was performed. The equivalent of 2% (*w*/*w*) of the gelling agent 12-HSA and 1% (*w*/*w*) of HA in acai oil was prepared. The mixture (12-HSA, acai oil, and HA) was heated to 85 °C for 30 min in a water bath and was stirred for 3 min by a magnetic stirrer. Subsequently, the mixture was cooled to room temperature and left for 24 h to obtain OG + HA [5].

#### 2.2.3. Evaluation of the Efficacy of the Organogel Containing Hyaluronic Acid

##### Preliminary Toxicology

1. Assessment of Cytotoxicity

Proficient human fibroblast cell lines (MRC5), maintained in cryogenic tubes immersed in liquid nitrogen, were thawed and cultured every 2 days in sterile 25 cm^2^ cell culture flasks. For each test, the cells were disaggregated in the flask with 0.25% (*w*/*v*) trypsin solution (Sigma-Aldrich^®^, St. Louis, MO, USA), neutralized with fetal bovine serum (DMEM, Sigma-Aldrich^®^) added in twice the volume of trypsin solution. The flask was centrifuged at 1500 rpm (687× *g*, Excelsa Baby 208 N^®^ centrifuge, Fanem) for 5 min, and the pellet was resuspended in DMEM. A Neubauer chamber was used to count the cells, and their viability was tested with 0.4% Trypan Blue dye.

The OG and OG + HA were dispersed in 20 µL of dimethyl sulfoxide (DMSO 0.5%). The solution was vortexed for 1 min, then aliquoted in different concentrations (10, 25, 50, 100, 250, and 500 µg/mL) in the laminar flow chamber for use in cell viability assays. The test was carried out using the MTT ([3-(4,5-dimethylthiazol-2-yl)-2,5-diphenyltetrazolium bromide]) assay. To carry out this assay, a method reported in the literature, with minor modifications, was used as follows [23,24,25]: 2.43 × 106 cells/well were incubated with different concentrations of OG + HA in sterile 96-well plates for 48 h. The culture medium was then discarded, and 20 µL of 0.05% MTT (Sigma-Aldrich^®^) was added to each well. Then, the plate was incubated for 3 h under 5% of carbon dioxide in an air-humidified incubator at 37 °C.

The negative control was performed with MRC5 cells, to which 0.5% of DMSO and 0.05% of MTT solution were added. After the incubation period, the supernatant was discarded by inverting the plate and drying on paper towel. Finally, for cell lysis to occur, 100 µL of DMSO (Sigma-Aldrich^®^) were added to each well. The plate was read at a wavelength of 570 nm, using a BioteK-Elx800^®^ microplate reader. Cell viability was expressed as the percentage of viable cells in relation to the control group (culture medium).

##### Ex Vivo Skin Permeation and Retention Study

1. Ex vivo permeation study

**Figure A1:**
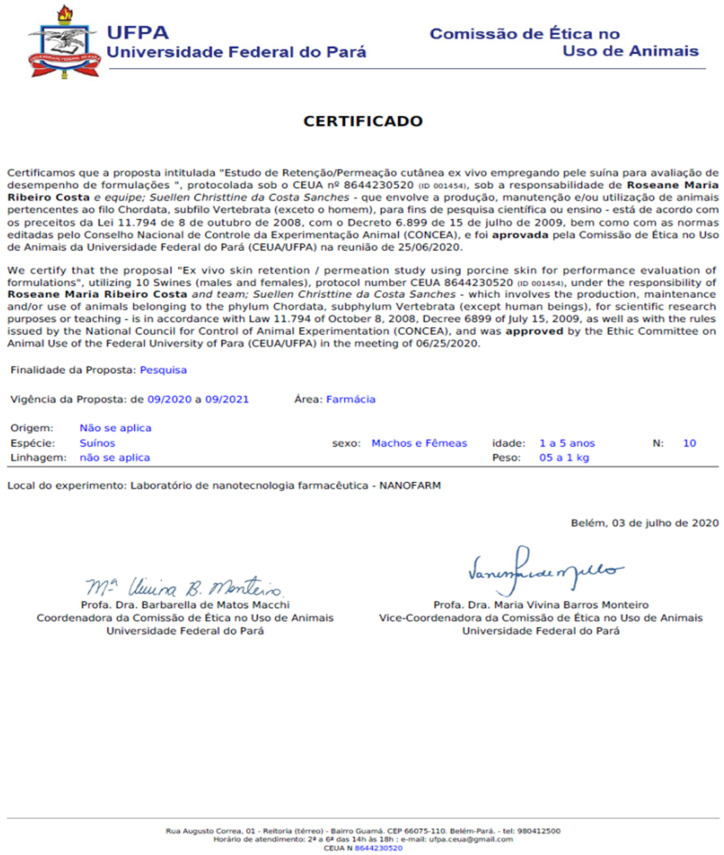
Ethics Committee for the use of Animals.

It should be noted that to carry out the ex vivo skin permeation and retention studies with pig ear membrane, the approval of the study by the Ethics Committee for the use of Animals in CEUA No. 8644230520 (attached in Appendix A Figure A1) was required. Biological membranes, obtained from the external region of the ear skin of young and albino pigs, were used shortly after slaughter. The pig ears were collected (parts with lesions, scratches, and stains were discarded), washed with purified water, and the excess water was removed with absorbent paper. The hair was cut off and dissected with tweezers and scalpel [26]. The dermatomized skins were wrapped in plastic film, aluminum foil, and kept in a freezer until use, not exceeding a period of 30 days [27].

For the permeation study, the Franz cell system with occlusion was used. The Franz cell system follows the bicompartmental model of diffusion cells, having two compartments, one containing the drug (donor compartment) and the other containing a solution in which the drug is soluble (receptor compartment), separated by a natural or synthetic membrane. The chosen receiving solution was phosphate buffer pH 7.2 [28]. The pig ear membranes were subjected to dermatomization to approximately 500 μm thick and 35 mm in diameter. In total, 200 µg of OG + HA (11.22 µg/mL of cross-linked HA) was added to the biological membranes. And 3 mL of receptor solution was maintained at 37 °C in a thermostated bath under constant stirring of 300 rpm, by a magnetic stirrer in the receiving compartment. The membranes with OG + HA were placed on the diffusion cell (donor compartment), keeping them in contact with the receiving compartment. The passage of HA through the membrane to the receptor compartment was monitored by analyzing samples of the receptor liquid, by collecting 1 mL of phosphate buffer at 1, 2, 4, 6, 8, and 24 h, with subsequent replacement of this volume at each sampling. The concentration of HA in the receiving compartment was determined by spectrophotometer at 530 nm (1800 UV-VIS, Shimadzu Europe Analytical Instruments, Norwood, MA, USA). HA quantification was performed in triplicate [28].

2. Ex vivo study of retention in the stratum corneum

After skin permeation study, the skins were maintained in the diffusion cells in order to evaluate the retention times at 2, 4, 6, 8, 24, 48, and 72 h. Excess organogel was removed from the donor compartment and washed with distilled water, then dried with absorbent paper. The skins were then subjected to the tape-stripping procedure. The skin was fixed on a Styrofoam support so that the exposure area could be subjected to extraction of the stratum corneum, in which 12 adhesive tapes were pressed and pulled over the skin. The first tape was discarded to remove excess product, and the remaining tapes were separately transferred to tubes containing 2 mL of methanol and 2 mL of distilled water. The tubes were vortexed for 1 min and then sonicated for 15 min in an ultrasound bath to disrupt the cells [29]. The concentration of HA in the supernatant was determined by the spectrophotometer at 530 nm. The experiment was carried out in triplicate.

3. Ex vivo study of retention in the epidermis and dermis

After removing the stratum corneum, the area of the skin exposed to permeation was fixed, cut, and minced with a scalpel and scissors. Retention times of 2, 4, 6, 8, 24, 48, and 72 h were evaluated. The skins were collected in tubes containing 2 mL of methanol and 2 mL of distilled water and then subjected to sonication for 30 min in an ultrasound bath to disrupt the cells [29]. The concentration of HA in the supernatant that was retained in the dermis and epidermis was determined by spectrophotometer at 530 nm. The experiment was carried out in triplicate.

##### Assessment of Skin Hydration

The pig skin membranes were used for the control (membranes without organogels) and observed after 24 h of retention. The OG, after 24 h of retention, and the OG + HA, at retention times of 2, 4, 6, 8, 24, 48, and 72 h, were analyzed by spectroscopy in the infrared region with Fourier Transform with reflectance attenuation (ATR-FTIR) under the following conditions: scan 32, resolution 4 cm^−1^, and in the range 4000–600 cm^−1^, using ATR on the IR Prestige Spectrometer 21 (Shimadzu^®^ Corporation, Kyoto, Japan) [30]. The water content calculation was carried out using the following Equation (1) [31]:

(1) $$Water\;content\left(\%\right)=\frac{Amide\;I}{Amide\;II}\;\times \;100$$

##### Thermal Analysis of Stratum Corneum

In total, 2.5 mg of the control pig skin membrane (without organogels) after 24 h of retention, of pig skin membrane with OG after 24 h of retention, and of pig skin membrane with OG + HA at retention times of 2, 4, 6, 8, 24, 48, and 72 h were weighed in aluminum crucible, hermetically sealed with a lid and were pierced. The analyses were performed under the following conditions: nitrogen atmosphere (50 mL/min) and heating rate of 10 °C/min in a temperature range of 25 to 200 °C. The enthalpy variation calculation was carried out using the TA 60w Shimadzu^®^ program [32].

### 2.3. Statistical Analysis

Mean and standard deviation (SD) were calculated. Statistical comparisons were made using an analysis of variance (ANOVA) and Tukey’s test using the GraphPad 5.0, adopting *p* < 0.0001 as the significance level.

## 3. Results

### 3.1. Physical–Chemical Characterization of the Acai Oil

Table 1 shows the physicochemical parameters of the acai oil used to evaluate the quality of the raw material chosen for preparing the organogels.

#### 3.1.1. Fatty Acids (FA) Profile

The profile of FA, found in the acai oil (Table 2), showed about 70% of unsaturated fatty acids, in agreement with the physicochemical results summarized in Table 1. The most abundant is oleic acid (59.41%), followed by linoleic acid (10.95%), palmitic acid (23.08%), and palmitoleic acid (3.69%). In addition, the values of 59.41% of monounsaturated fatty acid (MUFA), 14.64% of polyunsaturated fatty acid (PUFA), and 24.82% of saturated fatty acid (SFA) stand out.

#### 3.1.2. Attenuated Total Reflectance Fourier Transform Infrared Spectroscopy

**Figure 1:**
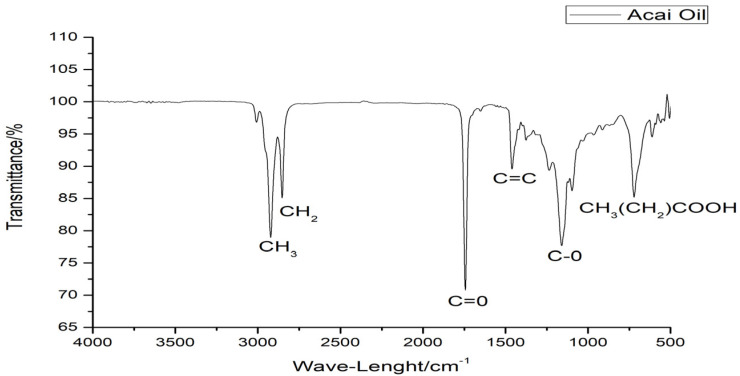
ATR-FTIR spectrum of acai oil.

The vibrational spectra below 3000 to 2750 cm^−1^ are characteristic of wide bands of methyl (-CH_3_), methylenic (-CH_2_), and ethyl (-CH) groups (Figure 1).

#### 3.1.3. Thermal Behavior

**Figure 2:**
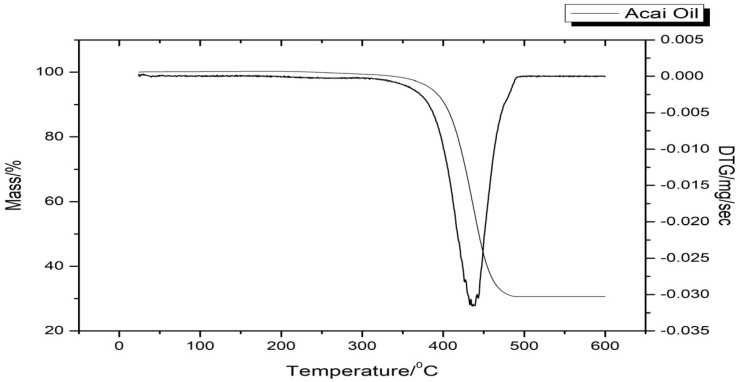
Thermogravimetric and derived thermogravimetry curves of acai oil.

The TG curve highlighted a mass loss in the temperature range between 384.71 and 451.57 °C, corresponding to the decomposition of the oil (Figure 2). The event is confirmed by the peak at about 440 °C in the DTG profile.

**Figure 3:**
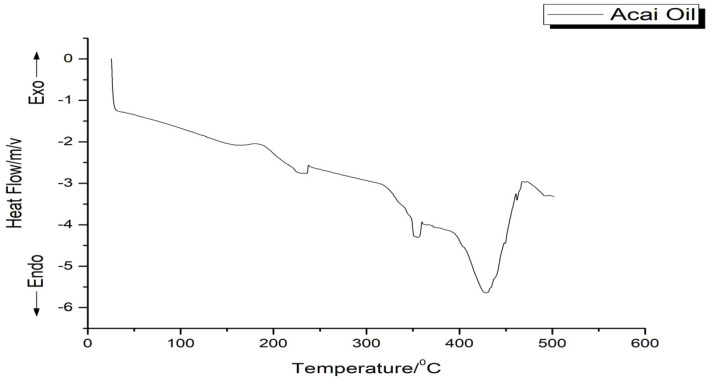
Thermogram of the acai oil.

The DSC thermogram of the oil exhibited three endothermic peaks. The first two are small events, occurring at around 230 °C (ΔH = 15.36 J/g) and about 350 °C (ΔH = 12.22 J/g), respectively. The third peak at around 440 °C (ΔH = 165.09 J/g) corresponded to the decomposition of the oil, confirming the observations in Figure 3.

#### 3.1.4. Quantification of Total Phenolic Content, Total Flavonoid Content, Total Carotenoids Content, and β-Carotene Content

Total phenolics exhibited the highest value in the acai oil, followed by total carotenoids, total flavonoids, and β-carotene (Table 3).

#### 3.1.5. Antioxidant Activity

The antioxidant activity of acai oil was evaluated by the three methods ABTS, DPPH, and β-carotene/linolenic acid system. Table 4 summarizes the antioxidant activities of acai oil.

The antioxidant activities of acai oil from the ABTS assay were positively associated with phenolic compounds (r = 0.965, *p* < 0.0001), total flavonoids (r = 0.951, *p* < 0.0001), carotenoids (r = 0.865, *p* < 0.0001), and β-carotene (r = 0.903, *p* < 0.0001). The antioxidant activities of acai oil from the DPPH assay were positively associated with phenolic compounds (r = 0.942, *p* < 0.0001), total flavonoids (r = 0.986, *p* < 0.0001), carotenoids (r = 0.882, *p* < 0.0001), and β-carotene (r = 0.928, *p* < 0.0001). The antioxidant activities of acai oil from the β-carotene/linolenic acid system assay were positively associated with phenolic compounds (r = 0.917, *p* < 0.0001), total flavonoids (r = 0.873, *p* < 0.0001), carotenoids (r = 0.982, *p* < 0.0001), and β-carotene (r = 0.898, *p* < 0.0001).

### 3.2. Evaluation of the Efficacy of Organogel Containing Hyaluronic Acid

#### 3.2.1. Preliminary Toxicity

##### Cytotoxicity Analysis

**Figure 4:**
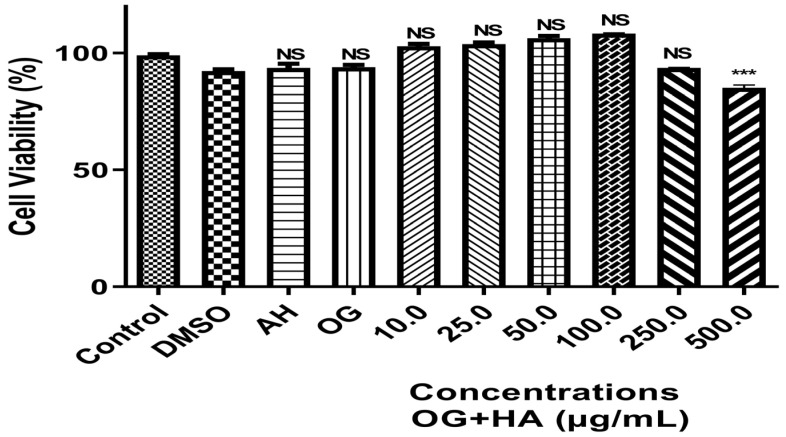
Effect of different concentrations of OG + HA on the viability of fibroblast cells of the MRC5 lineage incubated for 48 h at 37 °C in DMEM. Data were expressed as mean ± standard deviation (n = 3). ***: significative values for *p* < 0.0001 in relation to the control and other concentrations of OG + HA. NS: not significative values among themselves or compared to the control.

Cell viability analysis was evaluated in fibroblast cells of the MRC5 lineage incubated for 48 h with different concentrations of OG + HA (10–500 µg/mL). There was no decrease in cell viability at concentration range of 10–250 µg/mL. The organogel did not cause a cytotoxic effect on cells up to a concentration of 250 µg/mL. The IC50 value found was 300 µg/mL. From a concentration of 500 µg/mL, a significant difference was observed in relation to the control group (*p* < 0.0001) (Figure 4).

#### 3.2.2. Ex Vivo Skin Permeation and Retention Study

The study carried out at times 1, 2, 4, 6, 8, and 24 h on the OG + HA system demonstrated that HA was not able to permeate the skin membrane of the porcine ear, so the active ingredient could not reach the bloodstream.

**Figure 5:**
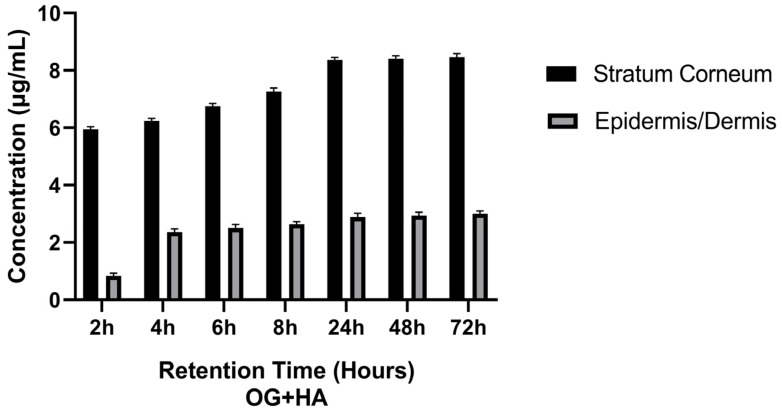
Effect of OG + HA on pig skin fragments after different retention times in stratum corneum and epidermis/dermis.

From the data reported in Table 5 and Figure 5, it can be observed that, as the contact time of the OG + HA formulation with the pig’s membrane increased, there was a proportional increase in HA in the stratum corneum and in the epidermis/dermis. After 6 h of formulation application, more than 80% of the HA was found in the stratum corneum and epidermis/dermis.

#### 3.2.3. Skin Hydration

**Figure 6:**
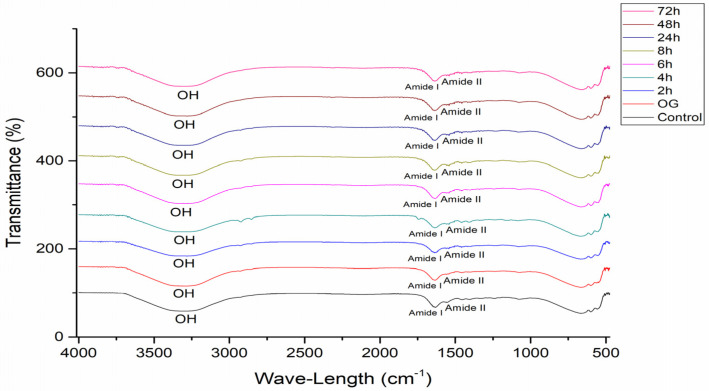
ATR-FTIR spectra of pig membranes after different retention times of OG + HA.

Figure 6 presents the ATR-FTIR spectra obtained in the study, highlighting the free hydroxyl groups (OH) bands, amide I, and amide II peaks.

**Figure 7:**
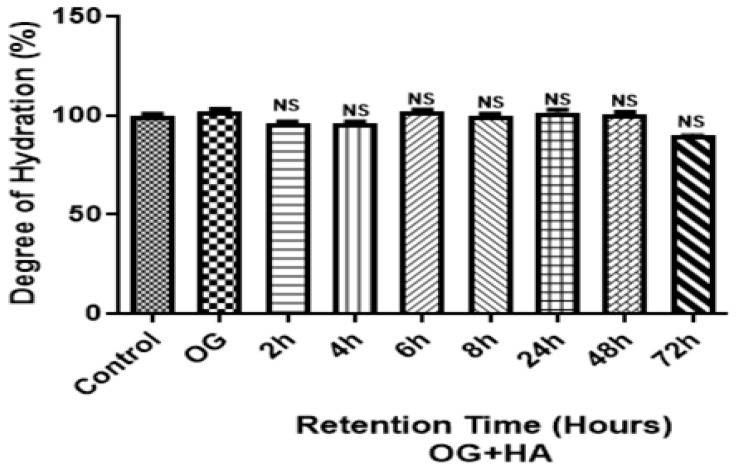
Effect of OG + HA on pig skin fragments after different permeation/retention times. Data were expressed as mean ± standard deviation (n = 3). NS: not significant values among themselves or compared to the control.

The hydration degree values at different times did not show significant differences (*p* < 0.0001) with respect to the control (fragment without organogel) (Figure 7). The skin hydration varied between 90.51 and 102.58%.

#### 3.2.4. Thermal Analysis of the Stratum Corneum

Table 6 summarizes the data of the pig skin fragment in contact with the OG + HA preparation for 72 h. The 6 h sample showed the highest enthalpy value (95.32 J/g). Then, after 6h of application of OG + HA, the pig skin membrane is thermally protected and hydrated, confirming the results of the ATR-FTIR study and correlating with the retention, data compatible with the general objective of the work, which is the preparation of a topical formulation.

## 4. Discussion

The quality control of the acai oil performed through the physical–chemical parameters (Table 1) of refractive index, density, kinematic viscosity, acidity index, peroxides, and saponification showed no change in their values compared to those reported in Codex Alimentarius Commission [12], Regulamento Técnico para Fixação de Identidade e Qualidade de Óleos e Gorduras Vegetais [33], and RDC No. 482/99 [34]. These results indicate that the oil under study presents standards of identity and quality suitable for use as a raw material in various sectors of industry. The value of refractive index is close to that established by the Codex Alimentarius Commission [12], which determines an index of around 1.45 for acai oil extracted from the pulp. From the density method, thus, it is observed that the evaluated acai oil had a density value in the recommended range (0.86–0.90) [11]. The ratio between the absolute viscosity and the specific weight of the fluid is called kinematic viscosity and is measured in stokes (mm^2^/s) [35]; in this study, the value was 43 mm^2^/s + 0.01. According to Regulamento Técnico para Fixação de Identidade e Qualidade de Óleos e Gorduras Vegetais [33], the recommended acidity index must not exceed 4 mg KOH/g for vegetable oils, and the value observed for the acai oil is in agreement, highlighting the quality of the oil under study. The peroxide index value confirmed the quality of the oil, as it is under the recommended value, which should be less than 10 meq/kg peroxide, according to Regulamento Técnico para Fixação de Identidade e Qualidade de Óleos e Gorduras Vegetais [33]. The saponification index result of the acai oil is compatible with the RDC No. 482/99 [34], which determined that the saponification index accepted is around 180–230 mg KOH/g.

Table 2 showed the profile of FA in acai oil. Among various types of FA, omega-3, omega-6, and omega-9 FA have been recognized to act as neuroactive molecules. About omega-9 FA, oleic acid is one of the most representative MUFA, which is usually considered to be beneficial in anti-inflammatory and vascular activities. Furthermore, oils rich in oleic acid spread better on the skin than oils with a high percentage of saturated acids. The consumption of PUFA plays an important role in humans by maintaining normal metabolism and health. Linoleic acid is the most highly consumed PUFA found in the human diet, and like all fatty acids, it can be used as a source of energy. It can be esterified to form neutral and polar lipids such as phospholipids, triacylglycerols, and cholesterol esters. As part of membrane phospholipids, linoleic acid functions as a structural component to maintaining a certain level of membrane fluidity of the transdermal water barrier of the epidermis [36,37].

As shown in Table 2, 23% of palmitic acid is present in the acai oil. Palmitic acid is frequent in human physiology and can be obtained from the diet and naturally through biosynthetic pathways, acting in reducing cholesterol, disrupting the homeostatic balance, and is implicated in different pathophysiological conditions, such as atherosclerosis, neurodegenerative diseases, and cancer [38,39]. The MUFA palmitoleic acid or palmitoleate can be found as a cis or a transisomer. The cis isoform has been associated with increased insulin sensitivity and decreased lipid accumulation in the liver. Transpalmitoleate is found in dairy products and partially hydrogenated oils and may be associated with favorable metabolic profiles and decreased incident diabetes [40,41].

The vibrational spectra (Figure 1) from 3000 cm^−1^ to 2750 cm^−1^ are characteristic of wide bands of methyl (-CH_3_), methylenic (-CH_2_), and ethyl (-CH) groups, similar to the spectral bands of buriti and Brazil nut oils [42]. Figure 1 showed a band in the high-intensity range of 1750 cm^−1^, being characteristic of the carbonyl group (C=O), characteristic of the steric group in long-chain fatty acids, such as those found in the FA profile of this oil, and 1710 cm^−1^ corresponds to the C=O stretches of carboxylic acid groups. Smaller spectral bands in the range of 1625–1500 cm^−1^ are observed with strong intensity and these bands could be due to the stretching vibration of C=C groups in the triglyceride structure. It is also possible to note a band around 1250 cm^−1^ to 1000 cm^−1^ that could be attributed to the acyl groups (C-O) [43]. The lowest observed spectral events in the 750 cm^−1^ range may be linked to the sequence of aliphatic fatty acid chains (CH_3_(CH_2_) COOH) [42].

The intensity of the spectral bands is compatible with the values found in the physicochemical tests, since these regions can be related to oxidative processes, causing evident connotation changes in the matrix with the degradation of carbon chains and production of compounds such as aldehydes, ketones, alcohols, carboxylic acids, peroxides, and hydroperoxides that progressively increase the degree of deterioration of the material [42].

The only event in the thermal study of acai oil, through the TG/DTG curve (Figure 2), is possibly correlated to the different sizes and numbers of unsaturation of fatty acids such as oleic acid and the presence of antioxidant substances resistant to temperatures above 200 °C. In addition, it is observed that the peaks are narrow and simple denoting the variation in the thermostability of the sample. This event can be best visualized by the decline in the baseline, as shown by the DTG curve, confirming the most severe decomposition in the temperature range of 384.71–451.57 °C, with 99.58% of mass loss; this result may be related to its FA composition, which contributes to its thermal stability.

The endothermic peaks present in the DSC thermogram of the acai oil (Figure 3) could be related to the decomposition of triacylglycerols that compose the oil, suggesting the evaporation and pyrolysis of FA. Furthermore, according to the ATR-FTIR results, the phenolic compounds, carotenoids, and flavonoids have in common the double bond that provides greater thermal stability, confirming the TG data, and may be related to greater efficiency in scavenging radicals [16]. Therefore, according to the results presented by thermal analysis, it was possible to observe the ability of the oil to resist at high temperature under a dynamic controlled atmosphere above 200 °C.

The levels of phenolic compounds and total flavonoids determined in acai oil correspond to what was observed in the literature and indicate its antioxidant potential in vivo to be used in food, cosmetic, or pharmaceutical formulations, as in the prevention and treatment of health disorders. The phenolic and flavonoid compounds are described as some of the main chemical components present in acai and responsible for its food, pharmaceutical, and cosmetic benefits. Only the presence of phenolic compounds does not characterize the antioxidant activity of a raw material, but indicates the presence of a group of substances with an antioxidant characteristic role, the flavonoids [44].

Vegetable oils and fats are predominantly composed of triglycerides, but in addition to triglycerides, traces of hydrophilic substances, such as flavonoids, can be found. These substances are normally found in very small quantities, due to their hydrophilicity, and are minor components of vegetable oils [3]. Sunflower, avocado, and canola oils have 0.06 mg RUT/g, 0.19 mg RUT/g, and 0.07 mg RUT/g [41], while acai oil presented 7.01 RUT/g (Table 3). This greater quantity of flavonoids in acai oil, when compared to canola, avocado, and sunflower oils, is probably due to the rich composition of flavonoids in the acai pulp, from which the oil is extracted.

Carotenoids, as a result of their conjugated polyene structure, play essential roles as accessory pigments in photosynthesis and the protection of plants and microorganisms against photooxidative processes. Several types of research indicate that the pro-vitamin A activity of some carotenoids has important applications both in the prevention of certain diseases and in the stimulation of the immune response at different levels and antioxidant action. The most abundant carotenoid in acai oil was β-carotene, the most efficient precursor of vitamin A, which is a highly lipid-soluble unsaturated polyene dye and antioxidant found in the plasma, besides its anticarcinogenic properties, aids in the absorption of nutrients and fat-soluble vitamins [45].

Factors such as the availability of light-intensive nutrients, climatic conditions, variety, harvest, and fruit maturation stage that affect plant phenolic compounds can lead to variations in phytochemical concentration. The presence of the studied bioactive compounds in acai oil is considered a positive characteristic, mainly for their antioxidant properties, which may have functions both in food technology, by increasing oxidative stability, and in human health; in addition, they can promote the high oxidative stability of the product resulting from the oil. These compounds prevent the auto-oxidation of oils and fats by providing their hydrogen to react with the free radicals formed in the initiation and propagation stages of auto-oxidation. Thus, they prevent radical formation in two ways: by scavenging them or by promoting their breakdown [45].

In this view, the ABTS method showed an antioxidant similar to the data observed in the literature. The DPPH analysis showed a 31.97% reduction in the DPPH radical in the presence of an antioxidant hydrogen donor. β-carotene/linolenic acid system demonstrated the result of the analysis, which is within the range of data observed in the literature between 40.14% and 74.659%, 42 indicating that the oil shows good activity by the methods tested.

These results highlight that acai oil can interact with different radicals, suggesting that it may have application as a functional food, reducing oxidative stress in the body, and/or a deteriorating situation resulting from the elevation of different reactive oxygen species (ROS). The variations in the content of bioactive compounds in acai, as well as in its antioxidant activity, can be attributed to the different places of origin of the fruits, to the way of obtaining the pulps, and to the conditions of transport and storage, besides the methodology applied for the determination of these compounds [46].

The results indicate that the antioxidant efficacy reflects the action of the bioactive compounds present in the oil in interrupting the oxidation initiation step. Thanks to its composition, acai has proven antioxidant activity against hydroxyl, peroxyl, and 2,2-diphenyl picrylhydrazyl radicals (DPPH) and also inhibits the oxidation of liposomes. In addition, among Amazonian fruits such as bacuri, abiu, cupuaçu, and graviola, the acai has the highest activity against 2,2-anizobis-3-ethylbenzthiazoline-6-sulfonic acid (ABTS) radicals. Currently, acai is used as an anti-inflammatory agent, reducing damage caused by oxidative stress and works against neurodegenerative diseases, even cancer. Bioactive compounds inhibit lipid peroxidation and lipo-oxygenation in vivo, allowing neutralization or sequestration of free radicals [44].

The model used for the cytotoxicity analysis was the MRC5 cell line, which consists of a population of fibroblastoid cells and was established from lung tissue. During the characterization phase of the MRC5 cells, it was observed the diversity of their possible applications, but also the property of this cell strain to remain in culture for long periods of time without showing signs of degeneration. MRC5 cell cultures, like other human fibroblast strains studied, have a limited capacity for expansion and the beginning of the decline of their proliferative potential occurs after reaching a level between 42 and 46 doublings of the cell population [47,48,49,50].

The result obtained does not show significant differences (*p* < 0.0001) between the groups analyzed (10 to 250 µg/mL) and the control, indicating that the OG + HA at these concentrations was not cytotoxic for this cell line, ratified by the IC50 value found. This means that this formulation does not release toxic substances into the medium that could prevent cell growth and adhesion impossible at the concentrations studied. The adhesion and cell viability of different cell types on substrates depends on the properties characteristics of these materials, such as wettability, presence of specific chemical groups, charge, roughness, and stiffness [47,51,52]. The data can be correlated with the chemical groups and the negative surface charge of the formulation.

The skin permeation test allows you to evaluate the amount of active ingredient that has permeated the skin and reached the bloodstream. The study carried out with the OG + HA organogel demonstrated that the preparation was not able to permeate the skin membrane of the pig ear so that the active ingredient could reach the bloodstream at times 1, 2, 4, 6, 8, and 24 h, since no significant HA value was quantified in the Franz cell receptor compartment. This result was expected due to the high molecular weight of HA, with the main resistance to the passage of compounds through the skin being caused by the compact structure of the stratum corneum. Furthermore, in topical dermatological products, it is desirable that the administered drug has a low flow rate into the bloodstream and high retention in the skin layers [53,54].

It was observed that, as the contact time of the OG + HA formulation with the pig membrane increased, there was a proportional increase in the concentration of the active ingredient in the stratum corneum and epidermis/dermis, with more than 80% of the HA released after 6 h of application of the formulation, as shown in Table 5 and Figure 5. For an anti-aging formulation, the results are favorable because in topical dermatological formulations, the active ingredient is not intended to reach the blood circulation, although it is important that it penetrates beyond the skin surface, i.e., into the stratum corneum, epidermis, and dermis. The permeation of the active ingredient contained in a cosmetic product must overcome some obstacles, such as the stratum corneum. However, when it comes to topical cosmetic products, it is desired that the active compound does not permeate the systemic circulation, but remains in the skin, and skin retention tests have demonstrated the ability of the active ingredient to remain in the skin layers for up to 72 h [54,55,56].

The causes of skin dryness are not yet perfectly understood; however, ATR-FTIR has been shown to be able to detect changes in the water content of the stratum corneum promoted by the use of OG + HA, according to the results presented previously. This method is widely used to measure skin hydration after the application of different cosmetic formulations [30,57,58]. Figure 6 shows the spectra obtained in the study, highlighting the free hydroxyl groups in the compound (OH), amide I, and amide II peaks [42,43]. Gloor [31] obtained the water content from the ratio between the amide I and amide II bands; amide I (1580–1720 cm^−1^) is related to the disordered stretching vibrational mode of the C=O molecule coupled with the vibrational modes of angular deformation of the NH molecule and the stretching of the CN molecule of the secondary structures of the protein; and amide II (1475–1580 cm^−1^) corresponds to the vibrational modes of angular deformation of the NH molecule and the stretching of the CN molecule. The higher this ratio, the greater the hydration, since the amide I band overlaps the water band [59].

The results showed no significant differences (*p* < 0.0001) (Figure 7) in the degree of hydration between the evaluated times of OG + HA in the pig skin fragments when compared to the control (fragment without organogel), demonstrating that the skin hydration varied in the range of 90.51–102.58%. However, it was observed that the highest percentage of water concentration was obtained after 6h of application of OG + HA, which correlates with the result of the retention study, which showed that more than 80% of the active ingredient was released after 6 h of application. Furthermore, it can be seen that the organogel retained a high percentage of water, over 90%, after 72 h of application of OG + HA, thus maintaining membrane hydration after a long period of time.

Table 6 provides two important data: the peak temperature (Tpeak) and the enthalpy. Since Tpeak corresponds to the maximum rate of evolution of the detected heating; however, it neither represents the maximum rate of change nor the end of the endothermic process [59]. Enthalpy represents the amount of heat required to evaporate the water present in the sample and/or to melt its crystalline structure. Regarding enthalpy, it can be said that the higher the enthalpy, the higher the amount of water present in the sample [60,61,62].

In terms of enthalpy, it is observed in Table 6 that the pork skin fragment that received the OG + HA preparation for 6h had a higher temperature at the beginning and end of thermal decomposition (41.53–205.29 °C) and the highest enthalpy value (95.32 J/g), indicating that the transition from the lamellar gel to liquid state, dehydration, and protein denaturation of the stratum corneum of the membrane occur at higher temperatures compared to other times of application of the organogel and the highest water content [63].

The results show that after 6 h of application of OG + HA, the pig skin membrane is thermally protected and hydrated, corroborating the results of the ATR-FTIR study and correlating with the retention, data compatible with the general objective of the work, which is the preparation of a topical formulation.

## 5. Conclusions

The physicochemical analyses carried out showed characteristic values of acai oil similar to those described in the literature and within the standards accepted by Brazilian and international legislation. The FA profile of the oil showed a predominance of MUFA and PUFA, such as oleic, linoleic, and palmitoleic acids, consistent with the values found by the lipid quality indices. The spectroscopic profile was corroborated by the well-defined bands corresponding to the functional groups present in the FA obtained by ATR-FTIR. Thermal analysis showed a profile typical of vegetable oils, with thermal degradation occurring above 200 °C.

The quantification studies revealed the presence of phenolic compounds, flavonoids, carotenoids, and β-carotene in significant concentrations to block the action of free radicals, as demonstrated by the antioxidant evaluation assays and statistical analysis. Thus, it can be concluded that the results indicate that the studied acai oil has sufficient physicochemical authenticity and antioxidant potential to be considered as a functional ingredient for application in food, pharmaceutical, and cosmetic fields.

The preliminary toxicological analysis of OG + HA using the cytotoxicity test showed that the formulation did not release toxic substances capable of causing cell death in the strain tested at different concentrations. In the in vitro permeation and retention study, the OG + HA presented a low fluidity profile for the bloodstream and high retention between the skin layers, which is consistent with the objective of a topical product, as the action of the active ingredient will be concentrated in the skin layers. ATR-FTIR and DSC tests on pig ear membranes showed that OG + HA is able to thermally protect the skin and maintain its hydration over a long period of time, highlighting that the highest water concentration was after 6h of application of the organogel.

Therefore, it is observed that OG + HA has potentially promised physicochemical, toxicological, and efficacy properties for application in the cosmetic areas. In addition, its composition uses a raw material from the Amazon region in a sustainable way and the preparation of the formulation does not require a complex infrastructure, making its cost–benefit an attractive factor for the industrial field.

## Figures and Tables

**Table 1:** Physical–chemical parameters of the acai oil (mean values ± standard deviation (n = 3)).

Parameters	Values
Refractive index	1.46 ± 0.01
Relative Density	0.90 ± 0.01
Kinematic Viscosity (mm^2^/s)	43 ± 0.01
Acidity index (mg KOH/g)	2.31 ± 0.02
Peroxide Index (meq H_2_O_2_ kg^−1^)	2.08 ± 0.01
Saponification Index (mg KOH/g)	209.13 ± 0.05

**Table 2:** Composition of fatty acids in acai oil (mean values ± standard deviation (n = 3)).

Nomenclature/Symbology	Percentage (%)	Retention Time
Oleic acid (C18:1)	59.41 ± 0.05	19.800 ± 0.03
Linoleic acid (C18:2)	10.95 ± 0.03	20.160 ± 0.01
Palmitoleic acid (C16:1)	3.69 ± 0.01	17.923 ± 0.02
Palmitic acid (C16)	23.08 ± 0.04	17.698 ± 0.03
Stearic Acid (C18)	1.74 ± 0.02	19.570 ± 0.02
MUFA	59.41 ± 0.01	-
PUFA	14.64 ± 0.01	-
SFA	24.82 ± 0.02	-

**Table 3:** Quantification of bioactive compounds of acai oil (mean values ± standard deviation (n = 3)).

Bioactive Compounds	Values
Total phenolics content (mg GAE/g)	72.08 ± 0.01
Total flavonoids content (mg RUT/g)	7.01 ± 0.03
Total carotenoids content (mg/100 g)	18.48 ± 0.05
β-carotene content (mg/100 g)	3.45 ± 0.01

**Table 4:** Antioxidant activity of acai oil (mean values ± standard deviation (n = 3)).

Antioxidant Activity	Values
ABTS free radical activity (µmol TE mL^−1^)	1242.89 ± 0.01
DPPH method (Trolox (TE)/g))	378 ± 0.02
β-carotene/linolenic acid system (%)	45.63 ± 0.03

**Table 5:** Retention of OG + HA in stratum corneum and epidermis/dermis (mean values ± standard deviation (n = 3)).

Time (Hours)	Concentration HA (µg/mL)		Concentration HA (µg/mL)	Concentration HA (%)
	Stratum Corneum	Epidermis/Dermis	Stratum Corneum + Epidermis/Dermis	Stratum Corneum + Epidermis/Dermis
2	5.88 ± 0.01	0.77 ± 0.05	6.65 ± 0.02	59.26 ± 0.03
4	6.17 ± 0.01	2.28 ± 0.04	8.45 ± 0.03	75.31 ± 0.02
6	6.69 ± 0.03	2.41 ± 0.03	9.10 ± 0.01	81.1 ± 0.05
8	7.17 ± 0.02	2.57 ± 0.01	9.74 ± 0.01	86.8 ± 0.01
24	8.3 ± 0.05	2.79 ± 0.02	11.09 ± 0.05	98.84 ± 0.04
48	8.33 ± 0.01	2.86 ± 0.02	11.19 ± 0.04	99.73 ± 0.03
72	8.38 ± 0.01	2.94 ± 0.01	11.32 ± 0.03	100 ± 0.02

**Table 6:** Enthalpy and peak temperatures of pig skin fragments from OG + HA.

Formulations	Thermal Event (°C)	Enthalpy (J/g)	Thermal Event (°C)	Enthalpy (J/g)
	**1st**		**2nd**	
Control	28.82–63.09	18.72	185.86–197.81	3.24
OG	30.25–74.82	29.39		
2 h	30.58–55.02	68.34	192.42–198.95	0.48
4 h	34.02–54.77	14.32	162.10–160.96	0.26
6 h	41.53–94.07	95.32	190.16–205.29	1.84
8 h	31.19–77.19	41.09		
24 h	29.65–67.69	5.26		
48 h	30.25–98.14	31.32		
72 h	28.34–62.87	17.23		

## Data Availability

All data are available in the article. They can be requested from the author for correspondence.
